# Indications and Outcomes of Nerve Reconstructions After Resection of Extremity Tumors: A Systematic Review

**DOI:** 10.1177/22925503251322527

**Published:** 2025-03-13

**Authors:** Bas Schuitema, Christianne Y. M. N. Jansma, J. Henk Coert, Enrico Martin

**Affiliations:** 1 Department of Plastic and Reconstructive Surgery, 8124 University Medical Center Utrecht, Utrecht, The Netherlands

**Keywords:** Tumor, functional outcomes, extremities, nerve reconstructions, systematic review, Tumeur, résultats fonctionnels, extrémités, reconstructions nerveuses, revue systématique.

## Abstract

**Objective:** Peripheral nerve injuries resulting from tumor resection are unusual, but occasionally unavoidable. It can result in serious morbidity in motor deficits, sensory deficits, and even chronic pain. Nerve reconstruction after tumor resection is possible and seems to have positive recovery outcomes. However, nerve reconstructions are rarely performed and clear outcomes of nerve reconstructions after tumor resection are missing. This review aims to create an overview of the indications and outcomes in these patients. **Methods:** A systematic review was performed in May 2024 in PubMed and Embase databases according to the PRISMA guidelines. Search terms related to “tumor” and “nerve reconstruction” were used. Studies evaluating nerve reconstructions (nerve graft, transfer or coaptation) after tumor resection were included. Tumors not located in the extremities were excluded. A qualitative synthesis was performed on all studies. Where possible, motor, and sensory grades were recalculated according to the Medical Research Council (MRC)-scale. **Results:** Fifty-nine articles were included for quality synthesis after full-text screening. A total of 90 patients were described of which 44 after resection of malignant tumors. Most patients improved in motor and sensory function after nerve reconstruction. In both benign and malignant tumors, 77% demonstrated functional recovery on the MRC scale of ≥3. Most of the patients, >80%, recovered to a protective sensation of S2 or higher. **Conclusion: **Nerve reconstruction after tumor resection can help recover both sensory and motor function and may avoid chronic nerve pain. Nerve reconstructions should therefore be considered in tumor surgery.

## Résumé

**Contexte :** Les lésions des nerfs périphériques secondaires à la résection de tumeurs sont inhabituelles, mais parfois impossibles à éviter. Il peut en résulter une importante morbidité en termes de déficits moteurs, déficits sensoriels et même douleur chronique. La reconstruction nerveuse après la résection d’une tumeur est possible et semble donner des résultats positifs. Toutefois, les reconstructions nerveuses sont rarement pratiquées et leurs résultats après résection tumorale font défaut. Cette revue vise à créer une vue d’ensemble des indications et des résultats chez ces patients. **Méthodes :** Une revue systématique a été menée en mai 2024 dans les bases de données PubMed et Embase en suivant les lignes directrices PRISMA. Les termes de recherche utilisés étaient liés à « tumeur » et « reconstruction nerveuse ». Les études évaluant les reconstructions nerveuses (greffe, transfert ou coaptation de nerf) après résection tumorale ont été incluses. Les tumeurs non situées dans les membres ont été exclues. Toutes les études ont fait l’objet d’une synthèse qualitative. Chaque fois que possible, les grades moteurs et sensoriels ont été recalculés selon l’échelle du MRC (Medical Research Council). **Résultats :** Cinquante-neuf articles ont été inclus pour une synthèse qualitative après évaluation complète du texte. Un total de 91 patients étaient décrits, parmi lesquels 44 avaient subi une résection de tumeurs malignes. La plupart des patients ont eu une amélioration des fonctions motrices et sensorielles après la réparation nerveuse. Dans l’ensemble des cas de tumeurs (bénignes et malignes), 77% des patients ont eu une récupération fonctionnelle ≥ 3 sur l’échelle du MRC. La majorité des patients (> 80%) a récupéré une sensibilité protectrice de niveau S2 ou supérieure. **Conclusion :** La reconstruction nerveuse après résection tumorale peut aider la récupération des fonctions motrices et sensorielles et peut éviter les douleurs chroniques d’origine nerveuse. Par conséquent, les reconstructions nerveuses devraient être envisagées dans la chirurgie tumorale.

## Introduction

Peripheral nerve injury after tumor resection is rare in benign tumors. In malignant tumors it can occasionally be unavoidable.^1,2^ Standard treatment in benign tumor resection is done with nerve preservation. In rare cases nerve resection should be considered^3,4^ Other nerve defects after benign tumor resection are often due to iatrogenic damage. In malignant tumors, nerve resection is sometimes the only option to achieve clear margins.^1,5^ Resection of nerves can lead to serious morbidity including motor deficits, sensory deficits, and even chronic neuropathic pain. Reconstructions following a nerve defect after tumor resection are possible, but are nevertheless not widespread used, likely due to barriers such as need for re-resection, unknown outcomes together with radiation and chemotherapy, complexity of managing extensive nerve gaps and a lack of literature in clear outcomes of nerve reconstructions after tumor resection.^6^ Preoperative collaboration with a reconstructive surgeon may help address these challenges and improve functional outcomes.

Nerve grafts are traditionally used to perform reconstructions. However, in recent years, nerve transfers have been increasingly performed in addition to nerve grafts following post-traumatic nerve injuries.^7^ A lack of literature on outcomes after nerve reconstruction creates a barrier among surgeons. In malignant tumors, radiotherapy and chemotherapy also can have a negative impact, in surgeons’ considerations for reconstructions.^6,8^

Overall, little is known about outcomes in patients with nerve defects after tumor resection. This review aims to create an overview of current literature and outcomes seen in this subset of patients with nerve reconstruction.

## Methods

### Literature Search

To identify all potentially relevant articles a systematic search was performed in both PubMed and Embase databases, according to the PRISMA guidelines (Preferred Reporting Items for Systematic Reviews and Meta-Analyses) in May 2024.^9^ A search string was built using terms related to tumor and nerve reconstruction. The exact search syntaxes for PubMed and Embase are shown in Supplementary Table S1. All relevant articles evaluating nerve reconstructions after extremity tumor surgery were included. By cross-referencing included articles, additional studies initially not included in our search were added. All nerve transfers, grafts and coaptations were considered nerve reconstruction. Exclusion criteria included: No extremities, no functional outcomes, and no Dutch or English. The initial review was conducted by two independent authors (B.S. and C.J.). Disagreements were solved through discussion, in which one additional author was involved (E.M.).

### Data Extraction and Synthesis

Data were extracted on individual patient and included the age of patients, gender, anatomical site of the tumor, injured nerve, tumor type, preoperative function, type of surgery, postoperative function, oncological outcome, size of nerve gap, type of reconstruction, graft type, and median follow-up. Patients with incomplete functional outcome data were excluded from the qualitative synthesis. Results were summarized with objective outcomes and subjective scales. Objective strength outcome measures are medical research council (MRC) muscle grade and use of ambulatory devices. Objective sensory measures are two-point discrimination or Semmes-Weinstein monofilament. The subjective measure includes The Musculoskeletal Tumor Society (MSTS) and the disability of the arm shoulder and hand (DASH) questionnaire. Patients without objective functional outcomes measuring, but with an accurate description of the functional abilities in follow-up, are translated to the minimum grade on the MRC scale.^10^ Patients without objective sensory outcome measuring but with an accurate description of sensory outcomes in follow-up, are translated to the minimal grade in MRC sensory scale. All patients were graded by two independent authors (B.S. and C.J.). Disagreements were solved through discussion, in which one additional author was involved (E.M.).

## Results

**Figure 1.**
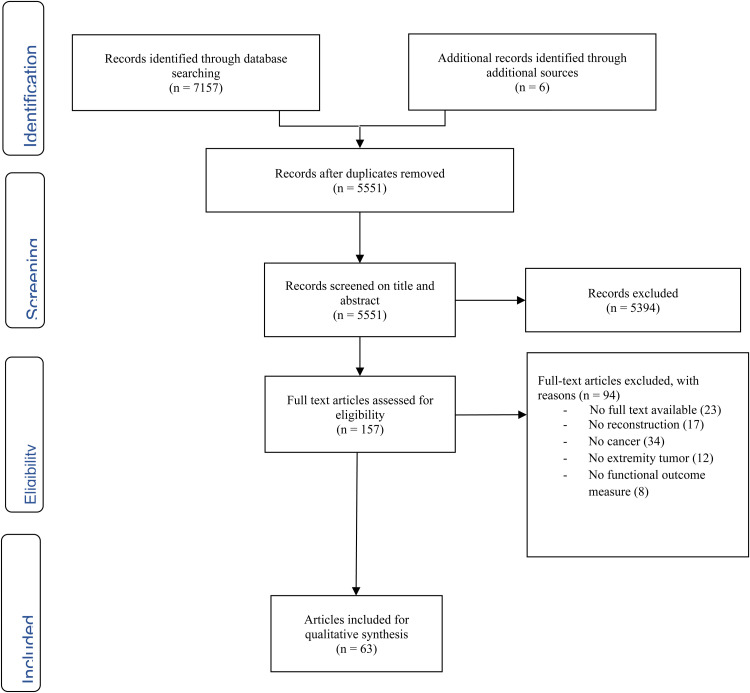
PRISMA flowchart.

After removing duplicates, a total of 4806 were identified in the PubMed and Embase database. Following title/abstract screening for eligibility, 153 potentially relevant articles were selected for full-text screening. A total 63 articles were included for quality synthesis after full-text screening (Figure 1). A total of 91 patients were described of which 44 after resection of malignant tumors. The mean age of patients 37 years old (range: 1 year to 67 years), of which 18 were children (Tables 1 and 2).

**Table 1.** Functional Outcomes Benign Tumors.

							Outcomes				
Pt.	Age	Tumor type	Nerve	Nerve gap	Type of reconstruction	Type of graft	Motor	Sensory	Add. Reconstr.	FUP	Add. surgery
**Upper extremity**											
1	9 Y	Perineurioma	N. radialis	3.5 cm	Graft	N. suralis	> 3	n.a.		6 m	
2	12 Y	Granular cell tumor	N. digitalis	2 cm	Graft	N. antebrachialis	4	4		1 Y	
3	11 Y	Intraneural nodular fasciitis	N. medianus	n.a.	Graft	N. suralis	5	4		2 Y	
4	3 Y	Perineurioma	N. medianus	2cm	Graft	N. suralis	0	n.a.	TT	3 Y	
5	2 Y	Perineurioma	N. medianus	5 cm	Graft	N. suralis	4	3		1 Y	
6	45 Y	Schwannoma	N. femoralis	15 cm	Transfer	N.a.	3–4	n.a.	VG	23 m	
7	3 Y	Lipofibroharmatoma	N. medianus	9 cm	Graft	N. suralis	3–5	n.a.		18 m	
8	35 Y	Lipofibroharmatoma	N. medianus	n.a.	Graft	N. suralis	4	2		3 Y	
9	66 Y	Schwannoma	P. brachialis	9 cm	Graft	N. suralis	5	4		7 d	
10	61 Y	Schwannoma	P. brachialis	8.5 cm	Graft	N. suralis	4	n.a.		3 m	
11	48 Y	Neurogenic tumor	P. brachialis	2.7 cm	Graft	N. suralis	>2	n.a.		9 d	
12	55 Y	Neurofibroma	N. ulnaris	6 cm	Graft	N. suralis	>3	n.a.		6 m	
13	30 Y	Harmartoma	N. medianus	2 cm	Graft	N. suralis	>3	2		1–3 Y	
14	12 Y	Harmartoma	N. medianus	3.5 cm	Graft	N. suralis	>3	2		1–3 Y	
15	34 Y	Harmartoma	N. medianus	15 cm	Graft	N. suralis	>3	2		1–3 Y	Thenar muscle atrophy
16	22 Y	Harmartoma	N. medianus	2 cm	Graft	N. suralis	>3	2		1–3 Y	
17	13 Y	Perineurioma	N. radialis	2.5 cm	Graft	N. suralis	1–4	n.a.		10 m	
18	13 Y	Neurofibroma	N. medianus	n.a.	Transfer	N.a.	4	3		7 m	
19	18 Y	BNST	N. suprascapularis	5.7 cm	Graft	N. suralis	3	n.a.		10 m	
20	30 Y	Glumos tumor	N. digitalis	n.a.	Repair	N.a.	n.a	3		6 m	
21	25 Y	Schwannoma	N. ulnaris	n.a.	Graft	N. suralis	>2	3		n.a.	
22	17 Y	Neurofibroma	N. radialis	4- 25 cm	Graft	N. suralis	n.a	1		n.a.	
23	29 Y	Neurofibroma	N. medianus	4- 25 cm	Graft	N. suralis	5	4		n.a.	
24	29 Y	Neurofibroma	N. ulnaris	4 cm	Graft	N. suralis	0	0		n.a.	
25	17 Y	Harmartoma	N. medianus	4- 25 cm	Graft	N. suralis	5	2		n.a.	
26	44 Y	Neurofibroma	P. brachialis	8 cm	Graft	N. suralis	0	0		n.a.	
27	7 Y	Neurofibroma	P. brachialis	n.a.	Graft	N. suralis	4	4		n.a.	
28	46 Y	Neurofibroma	P. brachialis	n.a.	Graft	N. suralis	3	2		1 y	
29	40 Y	Harmartoma	P. brachialis	n.a.	Graft	N. suralis	2	4		n.a.	
30	67 Y	Schwannoma	N. ulnaris	2 mm	Grafts	n.a	5	5	AG	n.a.	P.N.R.
31	24 Y	Lipoma	N. digitalis	n.a.	Repair	n.a.	5	5			
**Lower extremity**											
32	4 Y	Perineurioma	N. peroneus	n.a.	Transfer	N.a.	0	n.a.	TT	7 Y	
33	9 Y	Perineurioma	N. ischiadicus	6 cm	Graft	N.a.	0	n.a.	TT	8 Y	
34	24 Y	Schwannoma	N. femoralis	18 cm	Transfer	N.a.	4	n.a.		1 y 8 m	
35	55 Y	Neurofibroma	N. peroneus	7 cm	Graft	N. suralis	4	n.a.		7 Y	
36	27 Y	Schwannoma	N. femoralis	n.a.	Transfer	N.a.	3–5	n.a.		2 Y	
37	33 Y	Desmoid tumor	N. pectoralis	12 cm	Graft	N. suralis	4	n.a.		1 y	
38	52 Y	Fibromatosis	N. tibialis	15 cm	Graft	N. suralis	4	n.a.		1,5 Y	
39	14 Y	Perineurioma	N. femoralis	n.a.	Transfer	N.a.	3	n.a.		18 m	
40	27 Y	Harmartoma	N. femoralis	n.a.	Transfer	N.a.	5	n.a.		14 m	
41	40 Y	Schwannoma	N. femoralis	12 cm	Graft	N. suralis	4	2		3,5 Y	
42	57 Y	Schwannoma	N. femoralis	n.a.	Graft	N. suralis	4	2		2,5 Y	
43	48 Y	Schwannoma	N. femoralis	7 cm	Transfer	N.a.	4	n.a.		34 m	
44	44 Y	Schwannoma	N. peroneus	4- 25 cm	Graft	N. suralis	0	0		n.a.	
45	53 Y	Schwannoma	N. peroneus	4 cm	Graft	N. suralis	3–4	n.a.	VG	4 Y	
46	31 Y	Schwannoma	N. femoralis	n.a.	Transfer	N.a.	4	n.a.		2,5 Y	

^a^ CTX = adjuvant chemotherapy, AG = Allograft, aRTx = adjuvant radiotherapy, BNST = Benign nerve sheath tumor, BT = brachytherapy, Cm = Centimeter, d = Days, DTM = dead trough metastasis, FUP = Follow-up, MPNST = Maligne peripheral nerve sheath tumor, MT = Muscle transfer, m = Months, N.a. = Not available, nCT = neoadjuvant chemotherapy, N = Nerve, NF1 = Neurofibromatosis type 1, PF = Palliative focus, P = Plexus, P.N.R. = partial nerve resection, TT = tendon transfer, Y = Year.

**Table 2.** Functional Outcomes Malignant Tumors.

							Outcomes					
Pt.	Age	Tumor type	Nerve	Nerve gap	Type of reconstruction	Type of graft	Motor	Sensory	Add. recon.	Chemo /radiation	FUP	Add. Surgery
**Upper extremity**												
47	46 y	MPNST	N. medianus	n.a.	Graft	N. suralis	4	n.a.	TT	aRTx	5,5 Y	
48	29 Y	Synovial sarcoma	N. medianus	n.a.	Graft	N. suralis	4	n.a.	TT	n + aCT	11 Y	
49	27 Y	Synovial sarcoma	P. brachialis	n.a.	Graft	N. suralis	5	n.a.		n.a.	2,5 Y	
50	40 Y	MPNST	N. medianus	n.a.	Transfer	n.a.	3–5	n.a.	TT	aRTx	2 Y	
51	63 Y	Melanoma	N. suralis	n.a.	Transfer	n.a.	5	2	MT	n.a.	12 m	
52	52 Y	Melanoma	N. suralis	n.a.	Transfer	n.a.	5	2	MT	n.a.	12 m	
53	30 Y	MPNST	N. medianus	7 cm	Graft	n.a.	4	2		BT	2 Y	
54	33 Y	Liposarcoma	N. ulnaris	10 cm	Graft	N. suralis	3–4	3		n.a.	1 Y	
55	51 Y	Synovial sarcoma	N. medianus	10 cm	Transfer	n.a.	4	2–3	TT	aRTx	1 Y	
56	28 Y	Rhabdomyosarcoma	N. medianus	12 cm	Graft	N. femoralis	3–5	3		n.a.	2,5 Y	
57	65 Y	Breast carcinoma meta	N. ulnaris	n.a.	Transfer	n.a.	3–4	4		n.a.	2 Y	
58	24 Y	Synovial sarcoma	N. medianus	4.5 cm	Transfer	n.a.	4	1–4		n.a.	15 m	
59	34 Y	Squamos cel carcinoma	N. peroneus	8–10 cm	Transfer	n.a.	n.a	3		n.a.	26 m	
60	33 Y	Epithelioid sarcoma	N. medianus	n.a.	Transfer	n.a.	4	n.a.	TT	nCT	3 Y	
61	22 Y	MPNST	P. brachialis	8 cm	Graft/transfer	N. suralis	3–4	n.a.		nRTx	19 m	NF1
62	36 Y	MPNST	N. radialis	4- 25 cm	Repair	n.a.	n.a	4		n.a.	n.a.	
63	55 Y	Metastasis ductal carcinoma	P. brachialis	n.a.	Graft	N. suralis	0	0		n.a.	n.a.	Palliative focus
64	23 Y	Synovial sarcoma	N. medianus	n.a.	Graft	N. suralis	4	4		n.a.	7 m	
**Lower extremity**												
65	19 Y	Liposarcoma	N. femoralis	9 cm	Transfer	n.a.	4	n.a.		n.a.	1 Y	
66	24 Y	Epitheloid sarcoma	N. tibialis	11 cm	Graft	N. suralis	0	0		aRTx + aCT	8 Y	Grafts removed in secondary surgery
67	62 Y	Endometrial atypia	N. obturatorius	n.a.	Repair	n.a	5	5		n.a.	6 m	Damaged during surgery
68	60 Y	Endometrialad-enocarcinoma	N. obturatorius	3 cm	Graft	N. suralis	2–5	n.a.		aRTx	2 Y	
69	63 Y	Endometrium atypie	N. obturatorius	1 mm	Repair	n.a.	5	5		n.a.	6 m	
70	58 Y	Lymphadectomie	N. obturatorius	n.a.	Graft	N. suralis	5	n.a.		n.a.	n.a.	
71	65 Y	Liposarcoma	N. femoralis	12 cm	Transfer	n.a.	3	n.a.		n.a.	8 m	
72	36 Y	Squamos cel carcinoma	N. obturatorius	3 cm	Repair	n.a.	4	4		RTx	15 m	Biograft
73	18 m	Rhabdomyosarcoma	N. tibialis	n.a.	Transfer	n.a.	4	2		nCT + aCT	18 m	Prothese
74	75 Y	Liposarcoma	N. suralis	n.a.	Transfer	n.a.	n.a	n.a.		n.a.	14 m	
75	60 Y	Liposarcoma	N. gluteus minimus	n.a.	Transfer	n.a.	4	n.a.	MT	n.a.	3 y	
76	62 Y	MPNST	N. ischiadicus	14 cm	Graft	N. suralis	n.a.	n.a.		n.a.	3 m	
77	15 Y	Osteosarcoma	N. ischiadicus	14 cm	Graft	N. suralis	n.a	1–4		n.a.	18 m	Amputation
78	54 Y	MPNST	N. ischiadicus	13 cm	Graft	N. suralis	1–4	2		n.a.	2 Y	
79	50 Y	Papillary serous cystadenocarcinoma	N. obturatorius	n.a.	Repair	n.a.	5	4		n.a.	6 w	
80	n.a.	Leiomyosarcoma	N. femoralis	13 cm	Multiple	n.a.	2–5	n.a.	DTM	n.a.	n.a.	
81	54 Y	Sarcoma	N. peroneus	5 cm	Graft	n.a.	4	2	TT	n.a.	7 m	
82	44 Y	Synovial sarcoma	N. ischiadicus	15 cm	Graft	N. suralis	4	n.a.		nCT	1 Y	
83	41 Y	Fibromyxoid sarcoma	N. ischiadicus	19 cm	Graft	N. suralis	0–5	2		nRTx	2 Y	
84	19 Y	Sarcoma	N. ischiadicus	13 cm	Graft	N. suralis	0–5	2		n + aRTx AND n + aCT	8 m	
85	61 Y	Liposarcoma	N. ischiadicus	n.a.	Repair	n.a.	0–5	2		n.a.	42 m	
86	63 Y	MPNST	N. ischiadicus	8 cm	Graft	N. suralis	n.a.	n.a.		n.a.	15 m	
87	44 Y	Synovial cell sarcoma	N. Ischiadicus	15 cm	Graft	N. suralis	4	n.a.		nRtx + nCT	12 m	
88	43 Y	Sarcoma	N. Ischiadicus	13 cm	Graft	N. peroneus	2	0			25 m	
89	15 Y	Sarcoma	N. Ischiadicus	11,5 cm	Graft	N. peroneus	3	0		aCT	60 m	
90	62 Y	Sarcoma	N. Ischiadicus	12 cm	Graft	N. peroneus	4	1	DTM	aCT	10 m	
91	61 Y	Sarcoma	N. Ischiadicus	11 cm	Graft	N. peroneus	2	0			38 m	

^a^CTX = adjuvant chemotherapy, aRTx = adjuvant radiotherapy, BNST = Benign nerve sheath tumor, BT = brachytherapy, Cm = Centimeter, DTM = dead trough metastasis, FUP = Follow-up, MPNST = Maligne peripheral nerve sheath tumor, MT = Muscle transfer, m = Months, N.a. = Not available, nCT = neoadjuvant chemotherapy, N = Nerve, NF1 = Neurofibromatosis type 1, PF = Palliative focus, P = Plexus, TT = tendon transfer, Y = Year.

### Nerve Reconstructions After Benign Tumors

Nerve reconstructions after benign tumor resection were described in 46 patients (Table 1). Common reasons for nerve resection in benign tumors include severe pain, complex tumor, inadvertent resection, and risk for malignant degeneration.^11–14^ Sixty-seven percent of tumor resections were performed in the upper extremity. Thirty-four of the reconstructions were performed with a nerve graft. Nine reconstructions with a nerve transfer and 1 with a nerve repair. Seventy-seven percent of both children and adult patients with a benign tumor show a functional recovery to an MRC scale of ≥3.

In 25 patients’ sensory recovery has also been described. 84% of these patients recovered to a protective sensation (S2) or more. Six patients had no or poor recovery. One of these patients one year later died due a complication of NF1.^15^ Three patients with poor functional nerve recovery underwent tendon transfers to restore with a mean score of MRC 4.^16,17^ In two patients, there was no reason for failure. Especially 80% of adult patients show a functional recovery to an MRC scale of ≥3% and 93% of described sensory recovery reach a protective sensation (S2) or more.

### Nerve Reconstructions After Malignant Tumors

Nerve reconstructions after malignant tumor resection were described in 45 patients (Table 2). Most of the tumor resections were in the lower extremity (LE; 59%). Of the reconstructions 25 were performed with a nerve graft, 14 reconstructions are performed with a nerve transfer, and 6 with nerve repair. Seventy-six percent of the adult patients with a malignant tumor show a functional recovery on MRC scale of ≥ 3. Three patients were children, 2 of them received an amputation. One child showed a recovery of MRC scale of 3. Five patients had no or poor recovery. Two of these patients died through metastasis.^18,19^ One patient received an amputation. In one patient, nerve grafts were removed due to recurrence of tumor in the original nerve and graft.^20^ Nine patients received an additional muscle/tendon transfer, all of them achieved a functional recovery of on MRC ≥ 4. In 2 patients the reason for failure was not described.

In 25 patients’ sensory recovery has also been described. Of these patients, in >80% sensation recovered to a protective level (S2) or higher. Additional to the surgical resection, 14 patients received chemo- and/or radiotherapy. The mean functional outcome on MRC scale is 3. In our study, there no significant difference seen with patients who did not undergo chemo and/or radiotherapy.

### Nerve Grafts Versus Nerve Transfers

In this study, 65% of all cases were performed with a nerve graft. Twenty-five percent of the cases of the nerve defects reconstructed with a nerve transfer. The mean recovery at patient with a nerve graft is MRC 3. The mean recovery at patient with a nerve transfer is MRC 4.

### Children and Adult Outcomes

In this study 18 of the 91 patients are <18. Of the children diagnosed with a tumor, 89% were diagnosed with a benign tumor. Two out of 3 patients with a malignant tumor received an amputation. The mean motor outcome is children is MRC 3, however, the mean outcome in adults is MRC 4.

All cases with a tumor resection in children received minimal protective sensation (S2). Fifty percent had an almost full sensory recovery (S3/S4). Compared with adults, 13% have full loss of sensory function. All other adults regenerate minimal a protective sensation (S2).

### Upper Versus Lower Extremities

Fifty-six percent of the nerve reconstructions were performed in the upper extremity (UE), while 44% had a reconstruction in the LE. Nerve gaps in UE range from 2 cm to 25 cm. The mean functional outcome of the UE is MRC grade 3,5. Nerve gaps in LE range from 1 mm to 25 cm. The mean functional outcome of the LE is MRC grade 3,4. Differences are also minimal in sensory outcomes. The mean MRC sensory grade of the UE is 3 (2.6). The mean of the LE is 3 (2.5). On average, UE and LE restore sensibility to a protective sensation or more.

### Chronic Pain After Nerve Reconstructions

Pain after reconstruction was described in 35 patients. In 32 patients, there was no chronic pain following the nerve reconstruction. In 2 cases, chronic pain was remaining after reconstruction and for 1 case the pain decreased.

## Discussion

Reconstructions of nerves following tumor resection are described in both benign and malignant tumors. In both benign and malignant tumor resections, >80% achieved meaningful motor and sensory recovery after nerve reconstruction.

### Use of Nerve Reconstruction

Nerve damage can result in significant impairments in motor and sensory function, causing disruptions in daily activities and professional development. To minimize the effects of peripheral nerve damage, it is important to prevent it if possible. However, if it does occur, it will always lead to some degree of functional loss and/or neuropathic pain.

In cases of benign tumors, nerve resection was only performed in case of unavoidable circumstances. Common reasons for nerve resection in benign tumors include severe pain, complex tumor characteristics, inadvertent resection, and the risk of malignant degeneration. The decision to perform nerve resection in benign tumor must be carefully evaluated, considering both the benefits and drawbacks.^21,22^ Conversely, in malignant tumors, resection of adjacent nerves may be necessary to achieve optimal oncological outcomes. Most patients who underwent resection for malignant tumors in the extremities, including nerve resection, had soft tissue sarcomas. Primary literature in STS surgery mainly focuses on oncological outcomes, wide margins are often chosen to achieve optimal oncological outcomes.^23^ Wide margins can lead to the concomitant resection of critical healthy structures, potentially causing unnecessary functional loss. There is currently no consensus on the appropriate margins in malignant tumor surgery, as different guidelines result in varying values.^24,25^ A possible option to reduce margin without hand in on oncological outcomes is using radiotherapy. In soft tissue sarcomas, the indication for radiotherapy depends on various factors. It is primarily used when wide negative margins are not feasible or result in significant adverse effects.^24,26^ Adjusting margins in combination with radiotherapy can help preserve critical structures and, if necessary, facilitate functional reconstruction, ultimately leading to better functional outcomes without a significant compromise in oncological outcomes. The effects of radiotherapy on nerve reconstructions have been poorly documented, leading to reluctance among surgeons to combine both therapies,^27^

In trauma surgery, nerve reconstructions are frequently used for traumatic nerve injuries. Compared to tumor surgery, in trauma surgery nerve reconstructions are more commonly used. Research shows that reconstructions lead to good functional outcomes in trauma surgery,^28–33^ Literature shows in trauma surgery 47% to 96% have recovery of MRC scale ≥3 after a nerve construction.^33–37^ Even in sensory recovery, 81% reach an MRC grading of ≥S3 after nerve reconstruction,^38,39^ The average recovery of function after nerve reconstruction does not seem to differ greatly between tumor (including any radiotherapy) and trauma surgery.

### Types of Reconstructions

Various techniques are available nowadays for restoring function in extremities after tumor surgery. In addition to nerve reconstructions, muscle transfers, tendon transfers, and free flap reconstructions are options to stimulate functional recovery of the extremity.

Outcomes of functional reconstructions depend on type of resected tissue and location. In order to restore optimal function, the choice of reconstruction should be dependent on tumor size, location, and structures lost during resection, including skin tissue. Combining different types of reconstructions can be beneficial for optimal functional recovery. This study describes 11 patients where multiple types of reconstructions were combined. In these patients, a nerve reconstruction was combined with either a tendon transfer or a muscle transfer. Each of these patients achieved a minimum MRC grade 4.

In extremity surgery specifically, the next step in reconstruction is the recovery of critical sensation in addition to motor function^40^ While motor function may be regained in tendon reconstructions, muscle transfers, or nerve reconstructions, the restoration of sensory function is only possible by restoring nerves or nerve transfers. Following nerve repair, 55% to 63% of patients achieve good to excellent sensory function,^41^ Although sensory function is essential for the proper functioning of an extremity, it is unclear what the minimum required level of sensory function is, in combination with motor reconstruction, to ensure proper functional use of the limb. However, minimal protective sensation is essential for the functional use of the foot to prevent pressure ulcers and equally important in the hands to protect against accidental burns.

This study found that 85% of patients regained a protective sensation (S2) after nerve reconstruction.

Several options exist for nerve reconstruction. Whenever primary repair is not possible, nerve grafting has traditionally been the standard of care,^7,31^ In addition to autologous nerve grafting, nerve repair with both cellular and acellular allografts has become a new option in recent years with promising results.^42,43^ Over the past decades nerve transfers, originally developed for brachial plexus palsies,^31^ have been gaining popularity. They benefit from closer proximity to their end targets, thereby reducing the time needed for muscle reinnervation.

Our results indicate that nerve grafts up to 10 cm can achieve good motor outcomes, with strength levels reaching MRC 3–4. However, larger nerve gaps often lead to poorer outcomes due to the slow regeneration process. Patients who underwent nerve transfer, regardless of gap length, achieved an average motor outcome of MRC 4. In such cases, nerve transfers are particularly beneficial, as they enable faster reinnervation, reducing muscle atrophy and preventing functional loss. Functional outcomes and morbidity outcomes of nerve transfer in trauma surgery are positive and correspond to results from tumor surgery,^31–33^ However, comparisons between nerve grafts and nerve transfers should be made with caution, given the heterogeneity of the reconstructed nerves. In certain cases, nerve grafts may have been selected because nerve transfers were not a viable option.

### Chronic Nerve Pain

Chronic pain after tumor surgery is often multifactorial and difficult to treat,^44^ About 30% to 40% of patients with a curative treatment of cancer have been estimated to have chronic pain,^44,45^ Nerve damage is a common cause of chronic pain, approximately 15% to 25% of chronic pain causes neuropathic pain.^45–47^ Patients with nerve injury and chronic pain, have a higher frequency and greater degree of motor and sensory dysfunction compared to patients without chronic pain.^48^

Both surgical and non-surgical treatment are possible to reduce chronic pain. Various medications, injections, radiofrequency ablation and cryoablation can be used for chronic pain management, but reliable evidence is limited.^44,49^ Targeted muscle innervation (TMR) and regenerative peripheral nerve interfaces (RPNI) are surgical interventions that have beneficial outcomes in reducing pain. Beside TMR and RPNI, nerve reconstruction can reduce chronic pain.^48^

Chronic pain after nerve reconstruction is infrequently documented in the literature. Pain was reported in 35 of 84 patients in this study. For 2 patients, pain remained after reconstruction, and for 1 patient, the pain decreased. In all other cases, there was no pain after the after reconstruction. These outcomes suggest a possible positive influence of nerve reconstruction on prevention of chronic pain as an addition to motor and sensory recovery. However, more research needs to be conducted to gather the significant outcomes of this.

### Strengths and Limitations

Results of our study remain limited because of the low level of evidence of case reports and small case series. Functional outcomes were described in different (subjective) ways. Combined with the many different types of tumors and types of reconstructions, this makes comparisons complex. Additionally, in many of the included studies, the exact level at which the nerve resection was performed was not reported. The lack of precise descriptions makes it more difficult to estimate the expected nerve recovery, adding uncertainty to the interpretation and comparison of outcomes. Nevertheless, MRC grades for sensory and motor function could commonly be extracted or recalculated. Yet MRC grades may be lower than they are, as the MRC grading is based on the lowest described function achieved in the extremity. To make a comparison of functional outcomes more accurate, future literature must use similar objective measurement tools, for example, the MRC scale. Although it appears that there are good functional outcomes with nerve reconstruction after tumor resection, more research is needed to optimize patient selection and reconstructive algorithms.

The strength of this paper is that despite the sparsely available literature, we have shown that nerve reconstruction has the potential to improve the quality of life in patients with extremity tumors. It provides an impetus for further research in this area.

## Conclusion

Nerve reconstructions are viable reconstruction tools in tumor surgery as well. They provide the opportunity to restore lost motor function, sensory loss, and may even prevent chronic nerve pain, thus improving quality of life. Many options for reconstruction are available and a patient-tailored approach is best needed. As outcomes seem to be similar to nerve reconstruction in trauma cases, similar reconstructive algorithms could be applied.

## Supplemental Material

sj-docx-1-psg-10.1177_22925503251322527 - Supplemental material for Indications and Outcomes of Nerve Reconstructions After Resection of Extremity Tumors: A Systematic Review Supplemental material, sj-docx-1-psg-10.1177_22925503251322527 for Indications and Outcomes of Nerve Reconstructions After Resection of Extremity Tumors: A Systematic Review by Bas Schuitema, Christianne Y. M. N. Jansma, J. Henk Coert and Enrico Martin in Plastic Surgery
