# Supplementary material for: Indications and Outcomes of Nerve Reconstructions After Resection of Extremity Tumors: A Systematic Review
Source: Plast Surg (Oakv). 2025 Mar 13;34(2):266–77. doi: 10.1177/22925503251322527 (PMC11907512; doi:10.1177/22925503251322527)
Supplement: sj-docx-1-psg-10.1177_22925503251322527 - Supplemental material for Indications and Outcomes of Nerve Reconstructions After Resection of Extremity Tumors: A Systematic Review [file sj-docx-1-psg-10.1177_22925503251322527.docx]

**Supplementary table s1:** Search syntaxes for the Pubmed and Embase databases

**Search PubMed:**

(nerve[Title/Abstract] OR nerves[Title/Abstract]) AND (reconstruction[Title/Abstract] OR reconstructions[Title/Abstract] OR neurotization[Title/Abstract] OR remodeling[Title/Abstract] OR repair[Title/Abstract] OR graft*[Title/Abstract] OR crossover[Title/Abstract] OR transfer[Title/Abstract] OR transplantation[Title/Abstract] OR coaptation[Title/Abstract] OR "targeted muscle reinnervation" [Title/Abstract] OR "Regenerative peripheral nerve interface"[Title/Abstract] OR TMR[Title/Abstract] OR RPNI[Title/Abstract] OR "Nerve Transfer"[MeSH Terms]) AND (Carcinoma[Title/Abstract] OR tumor[Title/Abstract] OR tumors[Title/Abstract] OR cancer[Title/Abstract] OR cancers[Title/Abstract] OR malign*[Title/Abstract] OR sarcoma[Title/Abstract] OR neoplasm[Title/Abstract] OR neoplasia[Title/Abstract] OR neoplasias[Title/Abstract] OR "Neoplasms"[Mesh])

**Search Embase:**

(nerve:ti,ab OR nerves:ti,ab) AND (reconstruction:ti,ab OR reconstructions:ti,ab OR neurotization:ti,ab OR remodeling:ti,ab OR repair:ti,ab OR graft:ti,ab OR crossover:ti,ab OR transfer:ti,ab OR transplantation:ti,ab OR coaptation:ti,ab OR ‘targeted muscle reinnervation’:ti,ab OR TMR:ti,ab OR ‘Regenerative peripheral nerve interface’:ti,ab OR RPNI:ti,ab OR ‘Nerve Transfer’/exp) AND (Carcinoma:ti,ab OR tumor:ti,ab OR tumors:ti,ab OR cancer:ti,ab OR cancers:ti,ab OR malign*:ti,ab OR sarcoma:ti,ab OR neoplasm:ti,ab OR neoplasia:ti,ab OR neoplasias:ti,ab OR ‘Neoplasms’/exp) AND [article]/lim AND [embase]/lim
